# Clemastine Alleviates Depressive-Like Behavior Through Reversing the Imbalance of Microglia-Related Pro-inflammatory State in Mouse Hippocampus

**DOI:** 10.3389/fncel.2018.00412

**Published:** 2018-11-13

**Authors:** Wen-Jun Su, Ting Zhang, Chun-Lei Jiang, Wei Wang

**Affiliations:** ^1^Department of Stress Medicine, Faculty of Psychology and Mental Health, Second Military Medical University, Shanghai, China; ^2^Department of Navy Aviation Medicine, Faculty of Naval Medicine, Second Military Medical University, Shanghai, China

**Keywords:** depression, stress, inflammation, microglia, astrocyte, hippocampus, P2X7 receptor

## Abstract

**Backgrounds:** Abundant reports indicate that neuroinflammatory signaling contributes to behavioral complications associated with depression and may be related to treatment response. The glial cells, especially microglia and astrocytes in brain regions of hippocampus and medial prefrontal cortex (mPFC), are major components of CNS innate immunity. Moreover, purinergic receptor P2X, ligand-gated ion channel 7 (P2X7R) was recently reckoned as a pivotal regulator in central immune system. Besides, it was pointed out that clemastine, a first-generation histamine receptor H1 (HRH1) antagonist with considerable safety profile and pharmacological effect, may suppress immune activation through modulating P2X7R. Herein, we investigated the potential anti-neuroinflammatory effects of clemastine on chronic unpredictable mild stress (CUMS)-induced depressive-like behavior in a mouse model.

**Methods:** Male BALB/c mice were subjected to CUMS for 4 weeks, some of them were injected with clemastine fumarate solution. After the stress procedure, behavioral tests including Sucrose Preference Tests (SPTs), Tail Suspension Tests (TSTs) and locomotor activities were performed to evaluate depressive-like phenotype. Subsequently, expression of cytokines and microglia-related inflammatory biomarkers were assessed.

**Results:** In the present research, we found that clemastine significantly reversed both the declination of SPT percentage and the extension of TST immobility durations in depression mouse model without affecting locomotor activity. Also, we observed that clemastine regulated the imbalance of pro-inflammatory cytokines including interleukin-1 beta (IL-1β) and tumor necrosis factor alpha (TNF-α) in the hippocampus and serum of depressive-like mice. Additionally, clemastine significantly suppressed microglial M1-like activation specifically in the hippocampus, and also improved hippocampal astrocytic loss. Furthermore, clemastine downregulated hippocampal P2X7R without interfering with the expression of HRH1.

**Conclusion:** As a safe and efficient anti-allergic agent, clemastine could impressively alleviate stress-related depressive-like phenotype in mice. Further evidence supported that it was because of the potential function of clemastine in modulating the expression of P2X7 receptor possibly independent of HRH1, therefore suppressing the microglial M1-like activation and pro-inflammatory cytokines release in brain regions of hippocampus rather than mPFC.

## Introduction

Major depressive disorder (MDD), elicited by acute and chronic stress, is a prevalent, chronic and recurrent disease that affects over 350 million people worldwide and exacts a very large economic burden that is 10.3% of the total burden of disease (Smith, 2014). Recent reports indicate that neuroinflammatory cytokine signaling contributes to the pathophysiology of MDD (Treadway et al., 2015; Xu et al., 2015). Inflammatory biomarkers such as interleukin-1 beta (IL-1β), interleukin-6 (IL-6) and tumor necrosis factor alpha (TNF-α) were elevated in patients with depression who were free from any physical illness (Zunszain et al., 2013; Alcocer-Gómez et al., 2014). It has been demonstrated that, in animal models, inflammation mediates the development of depression- and anxiety-like behavior upon to stress stimulation (Liu et al., 2013; Pan et al., 2014). Furthermore, recent studies show that cytokine antagonist treatment could significantly improve mood and behavior in depressed patients with elevated inflammatory biomarkers (Raison et al., 2013; Weinberger et al., 2015). Taken together, inflammatory signaling contributes to behavioral complications associated with depression and may be related to the treatment response.

The glial cells, especially microglia and astrocytes, are the major sources of CNS innate immunity (Ransohoff and Brown, 2012). Over-activated microglia and subsequently activated astrocytes can release proinflammatory and cytotoxic factors, including TNF-α, IL-1β, IL-6, nitric oxide (NO) and reactive oxygen species (ROS; Graeber and Streit, 2010). Several studies suggest that microglia or astrocytes participate in the pathophysiology of depression within medial prefrontal cortex (mPFC) and hippocampus, the two major brain subregions implicated in mood disorders. Specifically, an increase in glial cell density has been observed in hippocampal subfields of CA and dentate gyrus in depressive subjects (Rajkowska and Miguel-Hidalgo, 2007). In addition, a decrease in astrocyte cell number and glial fibrillary acidic protein (GFAP) level in the hippocampus and PFC was also detected in patients with MDD (Rajkowska and Miguel-Hidalgo, 2007; Cobb et al., 2016). In animal studies, chronic unpredictable mild stress (CUMS) induces a reduction in astrocyte (GFAP^+^) density within the rat PFC (Banasr and Duman, 2007). Apart from that, microglial activation in hippocampus mediates chronic mild stress (CMS) induced depressive- and anxiety-like behavior (Wang Y.-L. et al., 2018). Inhibition of microglial activation by minocycline treatment leads to alleviations in IFN-α- or CMS-induced depression-like behaviors in rodents (Zheng et al., 2015; Wang Y.-L. et al., 2018). To date, accumulating evidence supports a crucial role of glial cells in mPFC or hippocampus in the brain inflammation and pathogenesis of MDD.

As to microglia specifically, it is recognized that they can be activated from resting condition into at least two different directions: M1 (classical) and M2 (alternative) polarization types, which are defined following the concept of macrophages. Generally, they usually act diversely on modulating the CNS immune system, even eliciting contradictive phenotypes (Butovsky and Weiner, 2018). In the past few decades, researchers gradually approved that M1-like microglia mainly express high levels of inducible NO synthase (iNOS) and proinflammatory cytokines including the aforementioned TNF-α, IL-1β, IL-6; while M2-like microglia often get high expression of arginase 1 (Arg1), chitinase 3-like 3 (Ym1) and also secrete immunosuppressive cytokines like IL-10 (Tang et al., 2018). Although it remains controversial owing to the oversimplification, this perspective of M1/M2 classification really helps in the evaluation of microglial status. For instance, both iNOS and Arg1 were found in microglia, they compete for the same substrate (L-arginine). Therefore, the counterbalance of these two enzymes can substantially affect the production of NO, a hallmark of oxidative stress, and indicate the pro- or anti-inflammatory profile of microglia. When it comes to practical applications, several researches have demonstrated that depressive-like mice induced by various stress protocols got prominent microglial activation, as well as secretions of inflammatory cytokines (Yirmiya et al., 2015). Moreover, it is postulated that the homeostasis of switchable M1/M2 microglia can not only reveal about the development and pathogenesis of CNS diseases, but also exert as a feasible therapeutic target towards diseases such as amyotrophic lateral sclerosis (ALS), Alzheimer disease (AD) and multiple sclerosis (MS; Butovsky and Weiner, 2018).

Meanwhile, it is well-known that in the nervous system, purinergic receptor P2X, ligand-gated ion channel 7 (P2X7R) is present on activated microglia (Bhattacharya and Biber, 2016) and astrocytes (Grygorowicz et al., 2016; Albalawi et al., 2017; Gao et al., 2017). There have been several studies showing that P2X7R activation could causally induce microglial release of IL-1β, TNF-α, IL-6, CCL2, CCL3 and CXCL2 (Bhattacharya and Biber, 2016). In astroglia, P2X7R activation also involved in priming IL-1β and NLRP3 inflammasome (Albalawi et al., 2017). These observations indicated that the P2X7 receptors are the important mediators of CNS inflammation. Furthermore, the P2X7R gene which is located on chromosome 12q24.31 has been identified as a susceptibility locus for affective disorders (Lucae et al., 2006). And recent studies have exhibited that P2X7R knockout could exert an antidepressant-like effect in mice (Basso et al., 2009; Boucher et al., 2011; Csölle et al., 2013). In accordance with the emerging evidence, P2X7R may play a pivotal role in depressive disorders and is also proposed as a prospective molecular target for therapeutic intervention in MDD.

Clemastine is a first-generation histamine receptor H1 (HRH1) antagonist with a favorable safety profile for use. Besides its well-known actions of antihistamine, recent studies found that clemastine can rescue schizophrenia-like behavior and social avoidance behavior in mice by enhancing myelination and oligodendrocyte differentiation (Mei et al., 2014; Li et al., 2015; Liu et al., 2016). In a placebo-controlled clinical trial, clemastine strongly reduced IL-6 and TNF-α production in peripheral blood macrophages and monocytes. This immunosuppressive function of clemastine was confirmed independent of H1R, because even in H1R-deficient mice, clemastine could successfully inhibit lipopolysaccharide (LPS)-dependent activation of macrophages (Johansen et al., 2011). In addition, clemastine reduces microgliosis, as well as modulates microglia-related inflammatory genes, such as P2X7R in SOD1-G93A mice (Apolloni et al., 2016a,b). In this study, we investigated the putative anti-neuroinflammatory effects of clemastine on CUMS-induced depression behaviors in animal model. We hypothesized that clemastine might ameliorate CUMS-induced depressive-like behaviors in mice by influencing cytokine release and modulating microglial or astrocytic activation to attenuate neuroinflammation in critical emotion-related subregions, such as the mPFC and hippocampus. The antidepressant-like effects of clemastine were evaluated by Sucrose Preference Test (SPT), Tail Suspension Test (TST) and Open Field Test (OFT). In addition, we assessed cytokines expression and microglia-related inflammatory genes in the mPFC and hippocampus.

## Materials and Methods

### Animals and Reagents

**Figure 1:**
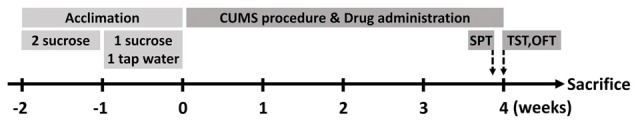
Schematic diagram of the research. Mice were allowed to acclimate for two consecutive weeks before stress protocol. After acclimation, they were divided into three groups (*n* = 10 for each group). At the end of chronic unpredictable mild stress (CUMS) procedure, behavioral tests were performed and then the animals were executed in the next day.

Male BALB/c mice aged 8 weeks were introduced from the Experimental Animal Center of Second Military Medical University (Shanghai, China). Before experimental manipulation, all mice were allowed to accommodate the new environment and 1% sucrose solution (weight/volume) for two consecutive weeks. In the first week, they were provided with two bottles of sucrose solution simultaneously. Afterward, one of the bottles would be changed with clean tap water in the next week (Figure 1). If not specifically clarified, mice would be allowed to acquire water and normal diet chow *ad libitum*. Light on/off cycle would be controlled in a 12 h/12 h shift, while the temperature of the animal room was kept steady at 22 ± 2°C. As for ethical considerations, the Animal Care and Use Committee of the Second Military Medical University authorized all procedures and treatments within the research.

After acclimation, the mice were assigned randomly to three groups (*n* = 10 for each group) for different treatments: Control group would be handled routinely, CUMS + Veh (Vehicle) group were injected with vehicle solutions, and CUMS + Cle (Clemastine) group were administered with clemastine solutions. Clemastine fumarate (Cat#: S1847, Selleckchem, Houston, TX, USA) were dissolved in DMSO and diluted in sterile 0.9% saline ahead of use (10 mg/kg/d intraperitoneally). Before each injection, the solutions would be mixed thoroughly and warmed up to 37°C.

### Chronic Unpredictable Stress Paradigm

The CUMS protocol is a widely used method to induce depressive-like behavior in rodents. Here, we applied a modified protocol which was based on our recently published data (Su et al., 2017). In brief, the operated mice would be raised separately and subjected to a series of stress stimulations for four continuous weeks. The schedule consisted of diverse stressful treatments including 10-min dry heat under 45°C, 5-min forced swimming in 4°C cold water, 30-min horizontal vibration, day-and-night inversion within 24 h, food or water deprivation for 20 h, wet bedding for 16 h, cage tilt for 12 h and constraint for 2 h. It is worth noting that only one type of stress would be conducted in each day, and successive use of the same stressor was not allowed. Drug and vehicle solutions would be administered at 17:00 every day throughout the CUMS procedure.

### Behavioral Analysis

Apart from recording body weights of the mice weekly, their behavioral parameters would also be determined before and after the CUMS agenda. In accordance with the published report, we performed three major behavioral tests at dark phase to evaluate the depressive-like phenotype: SPT, TST and OFT (Su et al., 2017). Considering that odor clue might interfere with the behavioral tendency, we used 75% ethanol to wipe the apparatus during TST and OFT manipulations, respectively.

Ahead of SPT, all the mice would be fasted for 20 h and then moved individually into new clean cages to acclimate for 1 h. Thereafter, two bottles in the same size and appearance, one filled with clean tap water and the other contained 1% sucrose solution, were provided simultaneously for each mouse. The consumptions of the fluids within an hour of test process were measured to calculate the sucrose preference proportion: sucrose solution intake/(tap water intake + sucrose solution intake)*100%.

As we know, SPT was used for evaluating anhedonia, while TST could reflect helplessness and desperation to some extent. In TST, a mouse would be hung upside down in the PHM-300 tail suspension chamber (MED Associates Inc., St. Albans, VT, USA). The chamber was able to transform stretching force into electric signals so that computer could record even a slight movement of the mouse, whose tail was stuck to a detecting hook. Since the first 1 min was allowed for adaptation, the subsequent 5 min of the whole 6-min test periods were valid for calculation of immobility. All movements with values under the threshold set at 0.75 would be determined immobile.

During the OFT, mice were allocated to the center of the trial box and thereafter moved freely in it. The box was in a 25*25*25 cm size and composed of detachable perforated acrylonitrile butadiene styrene copolymers walls (Cat#: RD1112-IOF, Mobile Datum Information Technology, Shanghai, China). Infrared cameras and a specific software helped with the recording and analyzing of moving traces throughout the 5-min test durations.

### Sacrifice and Sample Collection

After the accomplishment of CUMS protocol and serial behavioral analyses, experimental animals were euthanized. Blood sample would be collected in 1.5 mL Eppendorf tubes separately and allowed to coagulate at room temperature for 30 min, followed by centrifugation at 4,000 rpm for 15 min under 4°C. Supernatant serums were aspired carefully and transferred into 0.6 ml centrifugal tubes. Hippocampi and mPFC samples were dissected and isolated on ice. While sample collection were completed, specimen would be marked explicitly and flash frozen in liquid nitrogen, then preserved in −80°C refrigerator.

### Western Blotting

Tissues of hippocampus and mPFC were homogenized in cold RIPA lysis buffer (Cat#: P0013B, Beyotime Biotechnology, Nantong, Jiangsu, China). In order to prevent the protein degradation, lysis buffer was added with 1 mM PMSF (Cat#: ST506, Beyotime Biotechnology) and 10% PhosSTOP (Cat#: 04906845001, Roche, Indianapolis, IN, USA) before use. As previously described in our article (Su et al., 2017), enhanced BCA Protein Assay Kit (Cat#: P0010, Beyotime Biotechnology) was applied to determine protein concentration. After preparation of lysis samples, they were mixed thoroughly with 5× loading buffer (Cat#: P0015, Beyotime Biotechnology) and then heated to 100°C for 10 min. According to protein concentrations, samples containing equal amounts of protein were electrophoresed in SDS-PAGE gels (Cat#: PG112, EpiZyme, Shanghai, China) and transferred onto PVDF membranes (Cat#: ISEQ00010, Millipore, Billerica, MA, USA). After blocking in 5% skim milk at room temperature for 1 h, membranes were incubated with specific primary antibodies (Table 1) at 4°C overnight. In the next day, membranes would be washed and incubated with secondary antibodies (Table 1) at room temperature for 1 h. For visualizing and quantifying blotting bands, Odyssey Infrared Imaging System (LI-COR, Inc., Lincoln, NE, USA) and ImageJ Software (NIH, Bethesda, MD, USA) were utilized.

**Table 1:** Primary and secondary antibodies used in this research.

Primary antibody	Dilution	Host species	Cat#	Supplier	Secondary antibody	Dilution	Cat#	Supplier
Iba1	1:1,000	Rabbit	ab178846	Abcam
P2X7R	1:1,000	Rabbit	11144–1-AP	Proteintech				
IL-1β	1:800	Rabbit	ab9722	Abcam				
TNF-α	1:800	Rabbit	ab34674	Abcam	IRDye^®^ 800CW Goat anti-Rabbit IgG (H + L)	1:5,000	926–32211	LI-COR
iNOS	1:800	Rabbit	GTX130246	GeneTex				
Arg1	1:500	Rabbit	16001–1-AP	Proteintech				
HRH1	1:500	Rabbit	13413–1-AP	Proteintech				
GAPDH	1:2,000	Rabbit	10494–1-AP	Proteintech				
GFAP	1:1,000	Mouse	3670S	CST	IRDye^®^ 680RD Goat anti-Mouse IgG (H + L)	1:5,000	926–68070	LI-COR
IL-6	1:500	Goat	AF-406-NA	R&D Systems	IRDye^®^ 680RD Donkey anti-Goat IgG (H + L)	1:5,000	926–68074	LI-COR

### Enzyme-Linked Immunosorbent Assays (ELISA)

Concentrations of serum cytokines were measured using Mouse IL-1 beta Valukine ELISA Kit (Cat #VAL601, R&D Systems, Inc., Minneapolis, MN, USA), Mouse TNF-α ELISA kit (Cat #F11630, Westang, Shanghai, China) and Mouse IL-6 ELISA kit (Cat #F10830, Westang, Shanghai, China) according to the manufacturer’s instructions.

### Statistical Analysis

If not specifically clarified, the data was presented as mean ± SEM (standard error of the mean). One-way ANOVA with Tukey’s multiple comparisons tests were applied to analyze and compare continuous variables of FST, TST, OFT, ELISA and western blotting optical density calculation. Data of body weight change was analyzed by Two-way ANOVA of repeated measurements with Tukey’s *post hoc* tests. All the aforementioned analyses were performed using GraphPad Prism6 (GraphPad Software, Inc., La Jolla, CA, USA), and differences were defined statistically significant only when *p* < 0.05.

## Results

### Clemastine Ameliorates CUMS-Induced Depressive-Like Behaviors

**Figure 2:**
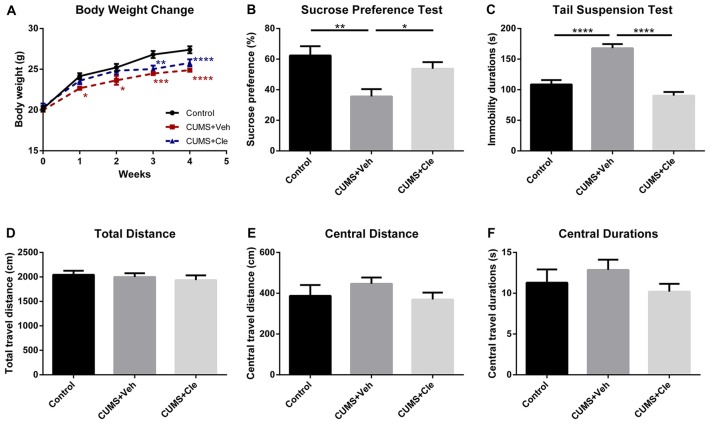
Body weight change throughout the stress procedure and behavioral analyses after the 4-week treatment.** (A)** From the first week of stress regimen, mice in CUMS + Veh group maintained less body weight than that of Control, whereas CUMS + Cle group did not show such a trend until the third week (*p < 0.05, **p < 0.01, ***p < 0.001, ****p < 0.0001 vs. Control). **(B)** At the end of week 4, lower sucrose preference ratio was detected in CUMS + Veh group (*p < 0.05, **p < 0.01). **(C)** In Tail Suspension Test (TST), immobility durations of CUMS + Veh group were longer than that of the other groups (****p < 0.0001). **(D–F)** As for analysis in locomotor activity, there is no significant difference among the three groups on all the displayed aspects (*n* = 10 for **A–F**).

We established the mouse model of depression induced by CUMS procedure for evaluating the effectiveness of clemastine on depressive-like behavior. From the third week of the CUMS procedure, we observed that the body weights of stressed mice maintained significantly less than that of the control group (Figure 2A). Along with that, the CUMS + Veh group mice exhibited a significantly lower sucrose preference percentage in SPT (Figure 2B), and significantly longer immobility time in TST (Figure 2C). During 4 weeks of clemastine treatment, the effect of CUMS procedure on body weight reduction maintained as compared with control group (Figure 2A). Nevertheless, clemastine treatment significantly improved the percentage loss of sucrose consumption (Figure 2B) and reversed the extension of immobility time in TST compared with CUMS + Veh group (Figure 2C). However, neither stress manipulation nor clemastine treatment interfered with the locomotive activity of the mice in OFT (Figures 2D–F).

### Pro-inflammatory Factors Levels in Hippocampus, mPFC and Serum

**Figure 3:**
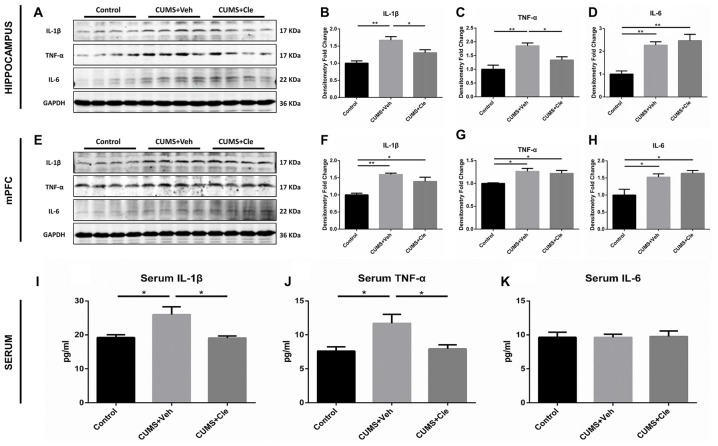
Detections of pro-inflammatory cytokines in hippocampus, medial prefrontal cortex (mPFC) and serum.** (A–D)** In the hippocampus, CUMS treatment resulted in higher levels of proinflammatory cytokines including interleukin-1 beta (IL-1β), tumor necrosis factor alpha (TNF-α) and interleukin-6 (IL-6). Continuous intervention of clemastine injection decreased the protein levels of both IL-1β and TNF-α. **(E–H)** In the mPFC area, manipulation of chronic stress coincidentally brought about elevated expressions of IL-1β, TNF-α and IL-6. Nevertheless, clemastine failed to modulate the upregulation of the aforementioned cytokines. **(I–K)** In serum samples, chronic mild stress (CMS) moderately elevated the concentrations of IL-1β and TNF-α rather than regulated IL-6 levels. Besides, clemastine remarkably cut down the amounts of IL-1β and TNF-α (*n* = 4 for **(A–H)** *n* = 5–6 for **(I–K)**, *p < 0.05, **p < 0.01).

In this study, pro-inflammatory cytokines IL-1β, TNF-α and IL-6 protein levels were significantly elevated in the hippocampus (Figures 3A–D) and mPFC (Figures 3E–H) in the CUMS + Veh mice compared with control mice. Additionally, we also detected moderate increases of IL-1β and TNF-α, but not alterations of IL-6, in serum specimens from CUMS + Veh group (Figures 3I–K). Clemastine administration for 4 weeks remarkably decreased IL-1β and TNF-α protein levels in hippocampus (Figures 3B,C) and serum (Figures 3I,J), but not in mPFC (Figures 3F,G) compared with CUMS + Veh group mice. Treatment with clemastine had no detectable effects on the expression of IL-6 in hippocampus, mPFC and serum samples (Figures 3D,H,K).

### Glial Activation Signatures in Hippocampus and mPFC

**Figure 4:**
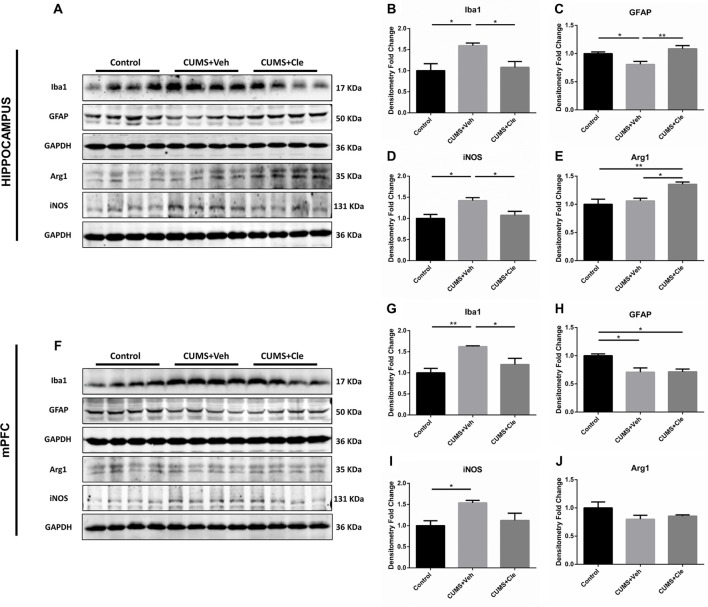
Detections of glial activation biomarkers in hippocampus and mPFC.** (A–E)** Western blot analysis of the hippocampal specimens indicated that, CUMS treatment inclined to increase the protein level of ionized calcium binding adaptor molecule 1 (Iba1) and inducible nitric oxide synthase (iNOS), as well as decrease the protein expression of glial fibrillary acidic protein (GFAP). Meanwhile, administration of clemastine could normalize these changes. Additionally, clemastine could upregulate Arg1 comparing to both Control and CUMS + Veh groups. **(F–J)** Similar with results of the hippocampus, chronic stress enhanced the expression of Iba1 and iNOS while suppressed the GFAP level in the brain region of mPFC. However, clemastine could only ameliorate the abnormality of Iba1 induced by CUMS. The expression of Arg1 remained unchanged in CUMS + Veh and CUMS + Cle groups compared with Control (*n* = 4 for **A–J**, *p < 0.05, **p < 0.01).

Western blotting was performed using cell-specific markers for microglia (Iba1, ionized calcium binding adaptor molecule 1) and astrocytes (GFAP) in hippocampus and mPFC following clemastine treatment, because these two cell types are main sources of inflammatory signals in the brain. Expression of microglia marker protein Iba1 was found to be increased in both hippocampus (Figures 4A,B) and mPFC (Figures 4F,G) compared with control group. However, expression of astrocyte marker protein GFAP was decreased in hippocampus (Figures 4A,C) and mPFC (Figures 4F,H) of this animal model. In hippocampus, clemastine treatment significantly reversed the changes in Iba1 protein (Figure 4B) and GFAP protein expression (Figure 4C) compared with CUMS + Veh group mice. Decreased Iba1 protein levels were also observed in mPFC (Figure 4G) after systemic clemastine treatment, while clemastine treatment did not alter the expression of GFAP protein in prefrontal cortex (Figure 4H) compared with CUMS + Veh group mice. In order to identify the polarization of activated microglia, iNOS and Arg1 were accepted as biomarkers of M1- and M2- like microglia, respectively. As is displayed, chronic stress stimulation led to overexpression of iNOS without affecting Arg1 in both subfields of hippocampus (Figures 4A,D,E) and mPFC (Figures 4F,I,J). However, 4-week injection of clemastine specifically downregulated hippocampal iNOS (Figures 4A,D) and also upregulated Arg1 in hippocampus (Figures 4A,E).

### P2X7 Receptor and Histamine Receptor H1 Expression in Hippocampus and mPFC

**Figure 5:**
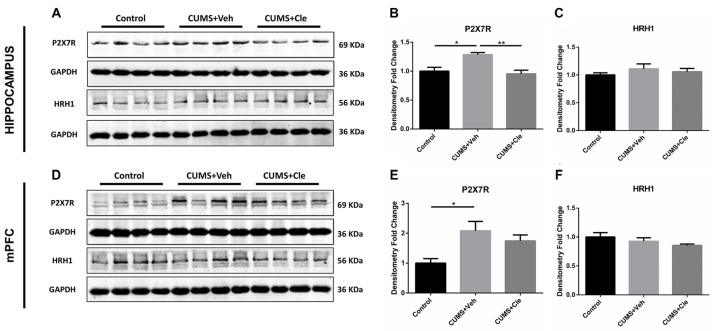
Detection of P2X7 receptor and histamine receptor H1 (HRH1) in hippocampus and mPFC.** (A–F)** CUMS treatment upregulated the protein expression of the purinergic receptor P2X, ligand-gated ion channel 7 (P2X7R) in both hippocampus and mPFC subregions. In the meantime, continuous intraperitoneal administration of clemastine protected mice from stress-induced upregulation of P2X7R in the hippocampus rather than mPFC. There is no statistical alteration of HRH1 expression in both subfields of hippocampus and mPFC, even when the mice were subjected to stress stimulation or clemastine injection (*n* = 4 for **(A–F)**, *p < 0.05, **p < 0.01).

To further investigate the antidepressant mechanism of clemastine, we analyzed both P2X7R and HRH1 expression in this study. As shown in Figure 5, significant increases of P2X7R expression were observed in hippocampus and mPFC of CUMS + Veh mice compared with control mice. Clemastine treatment significantly decreased P2X7R expression in hippocampus (Figures 5A,B) but failed to prevent the increase of P2X7R expression in mPFC (Figures 5D,E) of CUMS mice. The detection of histamine receptor would help with distinguishing whether the suppressive effect of clemastine on P2X7R was HRH1 dependent. Interestingly, we recognized that neither CMS nor clemastine could modify the expression of HRH1 (Figures 5A,C,D,F).

## Discussion

In the present study, we investigated the potential antidepressant-like effects of clemastine. Our results showed that clemastine significantly reversed the declination of sucrose preference and markedly reduced the extension of immobility time of TST in CUMS mice. In addition, we also observed that clemastine regulated the imbalance of pro-inflammatory cytokines IL-1β and TNF-α in both hippocampus and peripheral serum of depressive-like BALB/c mice. Subsequently, we found that clemastine remarkably suppressed M1-like microglial activation and ameliorated astrocytic loss mainly in the hippocampus. Moreover, clemastine downregulated hippocampal P2X7R independent of classical mechanism involving H1HR in hippocampus of CUMS mice.

Mounting evidence indicates that the MDD is associated with inflammatory alterations in emotion-related brain subregions, such as hippocampus and mPFC (Haroon et al., 2012; Bhattacharya et al., 2016; Goldsmith et al., 2016). Recent researches have suggested that inhibition of neuroinflammation might represent a novel mechanism of antidepressant treatment (Raison et al., 2013; Weinberger et al., 2015). Specifically, H1 receptor antagonist clemastine was convinced to possess immune suppressive properties and has been reported to participate in the pharmaceutical treatment of neuroinflammatory and neurodegenerative diseases (Johansen et al., 2011; Mei et al., 2014; Li et al., 2015; Apolloni et al., 2016b). According to a preclinical study, clemastine reduces IL-6 and TNF-α production in LPS-stimulated and *Listeria monocytogenes* (LM)-infected macrophages in a dose-dependent manner, and also suppresses those cytokines in serum of mice infected with LM (Johansen et al., 2011). Our present data manifested that 4-week CUMS procedure induced the imbalance of pro-inflammatory cytokines in the hippocampus and mPFC. We found that clemastine reversed the expression of the pro-inflammatory cytokines IL-1β and TNF-α but not IL-6 in the hippocampus and peripheral serum of CUMS mice. However, it failed to regulate those pro-inflammatory cytokines in mPFC, suggesting clemastine’s anti-neuroinflammatory effect by modulating cytokine release and hippocampal neuroinflammation might be involved in the antidepressant-like function. Meanwhile, we postulate that the anti-inflammatory function of clemastine differs between the hippocampus and mPFC may be attributed to heterogeneity of glial cells in specific brain subregions.

It is known that microglia and astrocytes are major sources of CNS inflammatory cytokines (Ransohoff and Brown, 2012). Many researchers have reported that the conditions of microglia and astrocytes were changed in different brain regions of depressed patients and animals (Banasr and Duman, 2007; Rajkowska and Miguel-Hidalgo, 2007; Yirmiya et al., 2015; Cobb et al., 2016; Wang Y.-L. et al., 2018). In our present study, the alterations of microglia and astrocytes in the brain were different. The expression of microglia maker Iba1 was increased in hippocampus and mPFC, being consistent with other reports of the proliferation and activation of microglia in hippocampus and PFC in unpredictable stress animal models (Kreisel et al., 2014; Pan et al., 2014; Wang B. et al., 2018). Further investigation confirmed that the activated microglia in depression mouse model were mainly M1-like polarized, which was characterized by abundant expression of iNOS and pro-inflammatory cytokines. In contrast, the expression of astrocyte marker GFAP was decreased in both hippocampus and mPFC, partly being consistent with the result of astrocytic loss in animal models subjected to chronic unpredictable stress (Heine et al., 2004; Czéh et al., 2006; Banasr and Duman, 2007). Considering kynurenine pathway (KP) was believed as a pivotal mediator of inflammation-induced depression, Wang B. et al. (2018) reckoned microglia activation facilitates the balance of KP towards a more depressive direction, while astrocyte mostly functions in a protective way. It seems that, to some extent, M1-like microglial activation leads to depressive-like behaviors.

Besides, clemastine has been shown to alleviate microgliosis, modulate microglia-related inflammatory genes, and enhance motor neuron survival (Apolloni et al., 2016a). In our study, clemastine treatment significantly downregulated the expression of Iba1 in both hippocampus and mPFC of CUMS mice, which suggests that clemastine hindered microgliosis. Specifically, being consistent with the result that clemastine reduced the expression of proinflammatory M1 marker NOX2 and simultaneously augmented M2 anti-inflammatory marker arginase-1 in SOD1^G93A^ mice (Apolloni et al., 2016b), we found that clemastine suppressed M1-like microglial activation as well as possibly improved M2-like polarization in hippocampus, therefore manifesting the putative effect of clemastine on regulating hippocampal M1/M2 switching. Compared with microglia, our results indicated that preventive treatment of clemastine protected astrocytes in hippocampus rather than mPFC of CUMS mice. Considering that Arg1, which exerts an anti-inflammatory and repair-facilitating influence via competing with iNOS for the breakdown of arginine, also exists in astrocytes (Liu et al., 2018), we cannot clearly distinguish which type of glia cell contributes more to the upregulation of Arg1 induced by clemastine. To some extent, microglia appeared to be primary in response to clemastine upon CUMS induced depressive-like behavior, while astrocytes might play a complementary role. Nevertheless, recent studies demonstrated that social stress resulted in release of inflammatory cytokines from monocytes which were recruited to the brain by microglia activation (Guillemin and Brew, 2004; McKim et al., 2018). Iba1, as microglial markers, is also expressed by other macrophage subtypes (Guillemin and Brew, 2004), thus we were unable to exclude the involvement of infiltrated macrophages in hippocampus and mPFC of CUMS mice. In line with these perspectives, further studies are needed to determine the exact origins and mechanisms in anti-neuroinflammatory effect of clemastine.

Meanwhile, a large body of evidence has been collected to support that P2X7 receptor may be pivotal in depression and mediates the IL-1β maturation (Zhang et al., 2016; Giuliani et al., 2017). Particularly recent studies have exhibited that P2X7 receptor knockout may have an antidepressant-like effect in mice (Basso et al., 2009; Boucher et al., 2011; Csölle et al., 2013). Moreover, in the nervous system, P2X7 receptors present on activated microglia (Bhattacharya and Biber, 2016) and astrocytes (Grygorowicz et al., 2016; Albalawi et al., 2017; Gao et al., 2017). In our study, the expressions of P2X7R were augmented in hippocampus and mPFC, which was partly consistent with previous findings (Pan et al., 2014). In several very recently published articles, researchers showed that P2X7 receptor antagonists including JNJ-55308942 and brilliant blue G (BBG) could modulate neuroinflammation and anhedonia in rodent models (Bhattacharya et al., 2018; Farooq et al., 2018). Concerned that Apolloni et al. (2016b) demonstrated that clemastine inhibited the increase of P2X7R expression in SOD1^G93A^ mice in lumbar spinal cord compared to vehicle group, we applied clemastine, a safe and effective clinical drug, as a potential P2X7 inhibitor. In our present study, we discovered that clemastine reversed the expression of P2X7R in hippocampus rather than mPFC of CUMS mice, partially being consistent with Apolloni’s results. However, Norenberg et al.’s (2011) study supported that clemastine could potentiate IL-1β secretion. In that study, they focused on the effect of clemastine on ATP-induced currents and [Ca^2+^]_i_ response rather than the expression of P2X7R protein in human blood-derived macrophages. They also suggest that clemastine is an extracellularly binding allosteric modulator of P2X7R that sensitizes P2X7R to lower ATP concentrations and facilitates its pore dilation and then augments the IL-1β release. Contradictorily, several previous studies pointed out that the modulation of P2X7R expression may through intracellular signaling pathway (Song et al., 2015; Yoshida et al., 2017). Therefore, we believe that these different functions of clemastine on P2X7R and pro-inflammatory cytokines releasing could be attributed to different detection methods, procedures, as well as test samples. Besides, histamine receptors may be irrelevant to the anti-inflammatory property of clemastine. In a research of HRH1-deficient mice, it was surprisingly demonstrated that the observed immune suppressive functions were HRH1 independent (Johansen et al., 2011). In consistence with that, we found that neither chronic unpredictable mild stress nor clemastine could alter the expression of HRH1. That is to say, HRH1 may be dispensable in the antidepressant-like action of clemastine via modulating microglial activation and suppressing pro-inflammatory cytokines.

Totally, we conclude the central immune imbalance which resulted from the activation of microglia and astrocytes may be responsible for depressive-like behavior induced by chronic unpredictable mild stress. When it shifts towards a M1-like proinflammatory polarization, various of deleterious cytokines would be secreted and then interfered with the normal function of central neurons and glial cells, leading to specific behavioral changes. To our knowledge, P2X7 receptor and downstream signaling might be a vital regulator in maintaining the aforementioned equilibration. As a safe and efficient anti-allergic agent, clemastine could significantly ameliorate stress-related depressive-like phenotype in mice. Further evidence supported that it was because of the potential function of clemastine in downregulating P2X7R independent of histamine H1 receptor, therefore suppressing the M1-like microglial activation and inflammatory cytokines release in brain region of hippocampus other than mPFC.

## Author Contributions

W-JS, TZ and WW wrote the manuscript and participated in all aspects of the research procedure. WW and C-LJ were involved in the experimental design and provided the funding, as well as the interpretation of the manuscript.

## Conflict of Interest Statement

The authors declare that the research was conducted in the absence of any commercial or financial relationships that could be construed as a potential conflict of interest.

## Abbreviations

Arg1: arginase 1
CUMS: chronic unpredictable mild stress
GFAP: glial fibrillary acidic protein
HRH1: histamine receptor H1
Iba1: ionized calcium binding adaptor molecule 1
IL-1β: interleukin-1 beta
IL-6: interleukin-6
iNOS: inducible NO synthase
LPS: lipopolysaccharide
MDD: major depressive disorder
mPFC: medial prefrontal cortex
NO: nitric oxide
OFT: Open Field Test
P2X7R: purinergic receptor P2X, ligand-gated ion channel 7
ROS: reactive oxygen species
SPT: Sucrose Preference Test
TNF-α: tumor necrosis factor alpha
TST: Tail Suspension Test.
